# Gastric Adenocarcinoma in a Patient with X-Linked Agammaglobulinemia and HIV: Case Report and Review of the Literature

**DOI:** 10.3389/fped.2016.00100

**Published:** 2016-09-23

**Authors:** Joud Hajjar, Sana Hasan, Lisa R. Forbes, Vagish Hemmige, Jordan S. Orange

**Affiliations:** ^1^Section of Immunology, Allergy and Rheumatology, Department of Pediatrics, Texas Children’s Hospital, Baylor College of Medicine, Houston, TX, USA; ^2^Internal Medicine, Baylor College of Medicine, Houston, TX, USA

**Keywords:** X-linked agammaglobulinemia, Bruton’s agammaglobulinemia, gastrointestinal malignancy, *Helicobacter pylori*, campylobacter, human immunodeficiency virus

## Clinical Implication

Patients with primary immunodeficiency are at an increased risk of cancer. Our case and literature review indicate an association between X-linked agammaglobulinemia and gastrointestinal malignancy and suggest that screening for gastrointestinal malignancy should occur for patients with X-linked agammaglobulinemia and gastrointestinal symptoms, unexplained anemia, or atrophic gastritis.

X-linked agammaglobulinemia (XLA) is an X-linked inherited disease caused by a germline mutation in the *BTK* gene leading to Bruton’s tyrosine kinase deficiency, which results in the impaired development of B-lymphocytes and a subsequent lack of immunoglobulin production. Patients with XLA have an increased susceptibility to bacterial and viral infections, and multiple case reports have been published regarding an association between XLA and gastrointestinal (GI) malignancy.

Here, we describe a case of a 25-year-old man with XLA and HIV, who developed gastric adenocarcinoma. Previously reported cases of XLA and GI malignancy are also reviewed and summarized.

## Case Report

A 25-year-old African-American man was referred to our center for continuous management of his XLA and HIV. At the age of 2 years, he started to develop recurrent infections, and based on a positive family history of XLA in a maternal uncle and several cousins, he was diagnosed with XLA and was started on intravenous immunoglobulin (IVIG) replacement since his diagnosis. Genetic testing was not initially done; however, recent genetic testing revealed a novel pathogenic variant in BTK c.307 C > T p.Gln103Ter (Q103X). His course was complicated by several sinopulmonary infections requiring hospitalization. Due to high-risk sexual behavior, he was screened for Human Immunodeficiency Virus (HIV) and at the age of 23 years and was found to be positive for HIV with high viral load; viral serology was never obtained due to his baseline agammaglobulinemia. He was asymptomatic and was started on, highly active antiretroviral therapy (HAART) (elvitegravir/cobicistat/emtricitabine/tenofovir), which maintained his CD4 count >1000 cells/mm^3^ and an undetectable viral load.

At the age of 24 years, he developed recurrent *Campylobacter jejuni* bacteremia and cellulitis of the leg, which was treated with meropenem for 4 weeks, subsequent episode was treated with a combination of intravenous meropenem and amikacin for 6 weeks. Suppressive doxycycline therapy was recommended, but the patient declined. Despite high-dose IVIG (700 mg/kg every 3 weeks), he continued to have immunoglobulin (Ig)G troughs (400–560 mg/dL). His stool culture, ova and parasite microscopic examination, and *Giardia* PCR were negative.

The patient had chronic iron-deficiency anemia and positive occult blood in his stool; therefore, an esophagogastroduodenoscopy (EGD) and colonoscopy were performed. His colonoscopy with random biopsy was normal. However, EGD showed gastric inflammation with erosions and ulceration. A gastric biopsy showed poorly differentiated gastric adenocarcinoma, which was *her2/neu* negative. The stain for *Helicobacter pylori* was positive. From a clinical stand point, except for his recurrent cellulitis, the patient did not have any other symptoms such as abdominal pain, early satiety, or weight loss. His review of systems as well did not reveal any respiratory symptoms. Abdominal computed tomography (CT) was negative for enlarged lymph nodes. However, a 1.4-cm arterially enhancing lesion in the left hepatic lobe was detected using abdominal and pelvic CT, with at least two adjacent sub-centimeter enhancing foci and two hepatic lesions. Abdominal magnetic resonance imaging characterized the larger lesion as hemangioma and the smaller lesions as cysts. Chest CT showed a 5-mm nodule, thought to be a granuloma. However, repeat CT scan of the lower lungs did not identify any nodules. His lungs otherwise were clear with no evidence of bronchiectasis or any other interstitial process. Genetic testing for CDH1 gene mutations, which are associated with hereditary diffuse Gastric cancer, was negative. He underwent subtotal gastrectomy, lymphadenectomy, and Roux-en-Y gastrojejunostomy. His post-operative pathology showed moderately poor differentiation of the adenocarcinoma with a signet ring component that was primarily mucosal with focal submucosal invasion, 0/20 nodes, negative margins, and diffuse metaplasia as well as omentum negative. Staining was positive for *H. pylori*.

His final diagnosis was stage 1A gastric cancer. No further treatment was recommended, and he was placed under surveillance with no evidence of malignant recurrence. However, he subsequently developed one episode of *C. jejuni* bacteremia that was treated with intravenous antibiotics.

Esophagogastroduodenoscopy was performed 1 year later and was negative for any ulcerations or concerning signs of recurrence.

## Discussion

Patients infected with HIV are at increased risk of certain malignancies, including breast, anal, cervical, lung, colorectal, prostate, and hepatocellular carcinoma (1). However, gastric cancer has not been reported to have increased prevalence among HIV-infected patients. Our patient did not have an acquired immune deficiency syndrome (AIDS)-defining clinical condition. On the other hand, patients with antibody deficiency are at greater risk of lymphoma and GI malignancy (2). The increased incidence of stomach cancer in patients with common variable immunodeficiency disorders could be related to the high frequency of achlorhydria.

In our literature review of GI malignancy and XLA, we found a total of 15 cases of GI malignancy (5 cases of gastric adenocarcinoma, 9 cases of colorectal carcinoma, and 1 case of liver cancer) in addition to our reported case (Table 1).

**Table 1:** **Review of the literature regarding patients with gastrointestinal (GI) malignancy and X-linked agammaglobulinemia**.

Case	Country	Presenting GI symptoms	Histology	Anemia	IgG (mg/dL) on IVIG	Solid tumor	Stage	Age (years)	Outcome
Adachi et al. (3)	Japan	Diarrhea; frequent urge to defecate	NA	NA	95	Colorectal	Local	22	Alive
Sasi et al. (4)	Saudi Arabia	Loss of appetite, weight loss, diarrhea, right-sided abdominal pain	NA	NA	NA	Necrotizing enterocolitis of the colon	Mets	10	Dead
Bachmeyer et al. (5)	France	Vomiting, dysphagia	Chronic atrophic gastritis, negative HP	NA	235–450	Gastric	NA	26	NA
Staines Boone et al. (6)	Mexico	Phlebitis of the right leg, weight loss	Gastric ulcer	Megaloblastic	625	Gastric	End	30	Dead
Brosens et al. (7)	Netherlands	Malabsorption, weight loss	NA	NA	NA	Colorectal	Local	45	Alive
Brosens et al. (7)	Netherlands	Weight loss	Positive HP	NA	NA	Colorectal	Local	37	Alive
Chisuwa et al. (8)	Japan	NA	NA	NA	NA	Colorectal	NA	NA	NA
Kinlen et al. (2)	UK	NA	NA	NA	NA	Gastric	End	25	Dead
Lackmann et al. (9)	Germany	Sensory deficit in the left leg, B12 deficiency	Negative HP; atrophic gastritis	Megaloblastic	172	Gastric	Early	15	Alive
Lavilla et al. (10)	Spain	Vomiting, diarrhea, weight loss	Atrophic gastritis	Megaloblastic	479	Gastric	Mets	23	Alive
Our case	USA	Cellulitis of the leg	Positive HP	NA	400–560	Gastric	1A	25	Alive
Vajdic et al. (11)	Australia	NA	NA	NA	NA	Gastric	NA	45	NA
Van der Meer et al. (12)	Netherlands	NA	NA	NA	NA	Colorectal	NA	36	Dead
Van der Meer et al. (12)	Netherlands	Abdominal distension, cramps, diarrhea	Villous atrophy	Microcytic	600	Colorectal	NA	NA	Dead
Van der Meer et al. (12)	Netherlands	Diarrhea, abdominal cramps, weight loss	NA	NA	Undetectable	Colorectal	NA	NA	Dead
Van der Meer et al. (12)	Netherlands	Diarrhea, abdominal cramps	NA	NA	NA	Colorectal	NA	36	Dead

*IgG, immunoglobulin G; IVIG, intravenous immunoglobulin; HP, Helicobacter pylori*.

The median age of male patients was 30 years (range, 7–40 years), indicating early onset of GI malignancy in patients with XLA. In the general population, the average age at diagnosis is 69 years, according to the American Cancer Society, and more than 80% of diagnosed cases of colorectal cancer occur in patients older than 55 years, according to a US task force.

Our patient had *C. jejuni* bacteremia, gastric inflammation, and *H. pylori* infection. He continued to have low IgG levels despite increasing the IgG replacement dose and frequency. We looked for a similar pattern in previously reported cases. Nine of the 16 patients had IgG levels <700 mg/dL, and 6 of these patients had levels <500 mg/dL, despite IVIG replacement. Two of the reported cases were positive for *H. pylori*, two were positive for *C. jejuni* bacteremia, two were infected with *Giardia lamblia*, and a majority had chronic atrophic gastritis. We hypothesized that chronic gastritis, with or without *H. pylori* infection, results in chronic inflammation, which could increase IgG catabolism or GI losses.

The anemia and positive occult blood in the present patient prompted the GI workup that led to his diagnosis. Three of the previously reported patients had megaloblastic anemia, and one patient had microcytic anemia. Chronic gastric inflammation can lead to achlorhydria, which in turn leads to megaloblastic anemia; this might be linked with gastric carcinoma in patients with common variable immunodeficiency (2) or XLA (6).

Finally, we reviewed the literature for the presenting signs and symptoms that support a diagnosis of GI malignancy; 10 of the 16 reported patients with XLA, who were diagnosed with GI malignancy, presented with weight loss or abdominal complaints such as diarrhea, a frequent urge to defecate, abdominal distension, cramping, and vomiting, which likely initiated the GI workup and led to an early diagnosis of GI malignancy. While there are many intestinal complications of antibody deficiencies, this one should be kept on the differential owing to its potential severity and treatability.

In conclusion, our case and review of the previous literature indicate an increased risk of GI malignancy in patients with XLA. The fact that our patient was HIV infected could have increased his risk of malignancy in general, although medical literature does not suggest particularly increased the risk of gastric cancer among HIV-infected patients. We found the presence of *H. pylori* infection and megalobastic anemia to be associated with GI malignancy in XLA, and since those could be detected in non-invasive ways (via urea breath test and B12 levels, respectively) we suggest screening for those factors in all patients with XLA and if present, this should prompt physicians to initiate a workup for GI malignancy in those patients.

Finally, we would like to note that our patient developed gastric cancer at the age of 23 years, Lackmann et al. described a 10-year-old boy with XLA and gastric cancer (9), and all cases summarized in Table 1 were below the age of 40 years, pointing out to the early onset of gastric cancer in this pediatric-onset disease, and that long-term follow-up for such patients raise the awareness about complications to be sought in this disease. Further studies to evaluate the cost-effectiveness of early screening for GI malignancy in patients with XLA might be warranted.

## Author Contributions

Conception or design of the work: JH. Data collection: JH and SH. Data analysis and interpretation: JH. Drafting the article: JH and SH. Critical revision of the article: JH, JO, and VH. Final approval of the version to be published: JH, SH, LF, VH, and JO. Agreement to be accountable for all aspects of the work in ensuring that questions related to the accuracy or integrity of any part of the work are appropriately investigated and resolved: JH, SH, LF, VH, and JO.

## Conflict of Interest Statement

The authors declare that the research was conducted in the absence of any commercial or financial relationships that could be construed as a potential conflict of interest.
